# A Combination Antibiogram Evaluation for *Pseudomonas aeruginosa* in Respiratory and Blood Sources from Intensive Care Unit (ICU) and Non-ICU Settings in U.S. Hospitals

**DOI:** 10.1128/AAC.02564-18

**Published:** 2019-03-27

**Authors:** Laura Puzniak, Daryl D. DePestel, Arjun Srinivasan, Gang Ye, John Murray, Sanjay Merchant, C. Andrew DeRyke, Vikas Gupta

**Affiliations:** a Merck & Co., Inc., Kenilworth, New Jersey, USA; b Division of Healthcare Quality Promotion, Centers for Disease Control and Prevention, Atlanta, Georgia, USA; c Becton, Dickinson and Company, Franklin Lakes, New Jersey, USA

**Keywords:** *Pseudomonas aeruginosa*, antibiogram, antibiotic resistance, antimicrobial susceptibility, combination susceptibilities, intensive care unit

## Abstract

Pseudomonas aeruginosa is an important pathogen associated with significant morbidity and mortality. U.S. guidelines for the treatment of hospital-acquired and ventilator-associated pneumonia recommend the use of two antipseudomonal drugs for high-risk patients to ensure that ≥95% of patients receive active empirical therapy. We evaluated the utility of combination antibiograms in identifying optimal anti-P. aeruginosa drug regimens. We conducted a retrospective cross-sectional analysis of the antimicrobial susceptibility of all nonduplicate P. aeruginosa blood and respiratory isolates collected between 1 October 2016 and 30 September 2017 from 304 U.S. hospitals in the BD Insights Research Database. Combination antibiograms were used to determine *in vitro* rates of susceptibility to potential anti-P. aeruginosa combination regimens consisting of a backbone antibiotic (an extended-spectrum cephalosporin, carbapenem, or piperacillin-tazobactam) plus an aminoglycoside or fluoroquinolone. Single-agent susceptibility rates for the 11,701 nonduplicate P. aeruginosa isolates ranged from 72.7% for fluoroquinolones to 85.0% for piperacillin-tazobactam. Susceptibility rates were higher for blood isolates than for respiratory isolates (*P* < 0.05). Antibiotic combinations resulted in increased susceptibility rates but did not achieve the goal of 95% antibiotic coverage. Adding an aminoglycoside resulted in higher susceptibility rates than adding a fluoroquinolone; piperacillin-tazobactam plus an aminoglycoside resulted in the highest susceptibility rate (93.3%). Intensive care unit (ICU) isolates generally had lower susceptibility rates than non-ICU isolates. Commonly used antipseudomonal drugs, either alone or in combination, did not achieve 95% coverage against U.S. hospital P. aeruginosa isolates, suggesting that new drugs are needed to attain this goal. Local institutional use of combination antibiograms has the potential to optimize empirical therapy of infections caused by difficult-to-treat pathogens.

## Abstract (precis)

Pseudomonas aeruginosa is an important pathogen associated with significant morbidity and mortality. U.S.

## INTRODUCTION

Pseudomonas aeruginosa is a clinically significant pathogen frequently associated with severe invasive infections, including hospital-acquired pneumonia (HAP), ventilator-associated pneumonia (VAP), and bloodstream infections (BSI). In the United States, P. aeruginosa accounts for approximately 20% of cases of HAP/VAP (1), with a mortality rate of 40% (2). Although P. aeruginosa is a less prevalent cause of BSI, mortality rates are similar (38.7% crude mortality rate for nosocomial BSI caused by P. aeruginosa in U.S. hospitals) (3) to those for BSI caused by other pathogens (4). P. aeruginosa is challenging to treat due to a plethora of intrinsic and acquired resistance mechanisms and the formation of biofilms resistant to antibiotic penetration (5, 6). In recognition of the clinical significance of P. aeruginosa resistance, the Centers for Disease Control and Prevention (CDC) has categorized multidrug-resistant (MDR) P. aeruginosa as a serious threat to human health (7), and the World Health Organization (WHO) has designated carbapenem-resistant P. aeruginosa to be a priority 1 critical pathogen for the research and development of new antibiotics (8, 9). Because of the high mortality from HAP/VAP caused by P. aeruginosa, current U.S. and European guidelines recommend empirical combination therapy with two antipseudomonal antibiotics from different classes to cover Gram-negative bacteria in high-risk patients (10, 11). In the United States, this recommendation specifies the use of two antipseudomonal antibiotics from different classes for HAP patients who are at risk for a Gram-negative bacterial infection or mortality. Dual antipseudomonal therapy is also recommended for VAP patients with risk factors for antimicrobial resistance, including recent hospitalization or intravenous antibiotic use, or in units where >10% of Gram-negative bacterial isolates are resistant to an agent being considered for monotherapy (10). Similar recommendations concerning combination therapy have been made by a European HAP/VAP task force and the Surviving Sepsis campaign (11, 12). The U.S. guidelines for the treatment of HAP/VAP comment that the goal of combination therapy is to ensure that ≥95% of patients receive empirical therapy that is active against likely pathogens. Because of problems with low lung penetration, aminoglycosides are not recommended as P. aeruginosa monotherapy or as the sole antipseudomonal drug in combination regimens (10). Despite recommendations for the use of combination therapy in high-risk patients, Clinical and Laboratory Standards Institute (CLSI) guidelines, which influence antimicrobial susceptibility reporting at most U.S. hospital laboratories, do not include guidance on the use of combination antibiograms to evaluate possible therapies.

To provide further insights on the potential utility of combination antibiograms in evaluating antipseudomonal coverage, we used data from a large nationwide clinical database to assess *in vitro* antimicrobial susceptibility rates for nonduplicate P. aeruginosa respiratory or blood isolates in intensive care unit (ICU) and non-ICU settings in U.S. hospitals.

## RESULTS

Isolates from a total of 304 hospitals in the BD Insights Research Database that met the inclusion criteria were included in the primary analysis. Of the 11,701 nonduplicate P. aeruginosa blood or respiratory isolates, most (89.4%) were obtained from respiratory samples and about 44% were obtained from an ICU (Table 1). The distribution of pathogens among hospitals generally reflected the hospital characteristics of the BD Insights Research Database, which includes a higher proportion of larger, urban hospitals than the group of hospitals certified by the Centers for Medicare & Medicaid Services (see Table S1 in the supplemental material). Most P. aeruginosa isolates (95.5%) were collected at urban hospitals (Table 1), and approximately 70% (70.2%) of the isolates were reported by hospitals with more than 300 beds.

**TABLE 1:** Distribution of facilities and P. aeruginosa isolates^*a*^

Characteristic	% of facilities (*n* = 304)	Isolates	
		No.	% distribution
Hospital characteristics			
Urban or rural			
Urban	82.9	11,176	95.5
Rural	17.1	525	4.5
Teaching status			
Nonteaching	62.8	4,797	41.0
Teaching	37.2	6,904	59.0
Bed size			
>300	36.2	8,216	70.2
100–300	45.7	3,092	26.4
<100	18.1	393	3.4
CDC region			
South	46.3	5,785	49.4
Midwest	29.3	3,293	28.1
West	13.2	1,428	12.2
Northeast	13.2	1,195	10.2
Isolate source			
Tissue			
Respiratory	NA	10,465	89.4
Blood	NA	1,236	10.6
ICU status			
Non-ICU	NA	6,544	55.9
ICU	NA	5,157	44.1
Infection onset			
Hospital	NA	6,445	55.1
Admission	NA	5,256	44.9

a Data are for 11,701 isolates. NA, not applicable; CDC, Centers for Disease Control and Prevention; ICU, intensive care unit.

### P. aeruginosa antimicrobial susceptibility to single agents.

The overall susceptibility of P. aeruginosa isolates ranged from 72.7% for fluoroquinolones (FQs) to 85.0% for piperacillin-tazobactam (Table 2). For all of the backbone antibiotics, susceptibility rates were higher for blood isolates than for respiratory isolates (*P* < 0.05 for all comparisons). None of the single-agent options attained a susceptibility of 95% for blood or respiratory P. aeruginosa isolates.

**TABLE 2:** Susceptibility by ICU status, source of P. aeruginosa isolate, infection onset, and hospital characteristics^*a*^

Characteristic	No. of isolates	% susceptibility												
		ESC			Carb			TZP			AG		FQ	
		Single agent	FQ combo	AG combo	Single agent	FQ combo	AG combo	Single agent	FQ combo	AG combo	Single agent	FQ combo	Single agent	AG combo
All	11,701	79.0	86.1	90.0	79.3	85.0	90.2	85.0	90.5	93.3	82.5	87.7	72.7	87.7
ICU status														
ICU	5,157	77.9	85.3	90.7	76.7	83.1	90.1	83.7	89.8	93.4	84.2	88.0	72.2	88.0
Non-ICU	6,544	79.8	86.7	89.5	81.4	86.5	90.3	86.0	91.1	93.1	81.1	87.5	73.1	87.5
Source														
Respiratory	10,465	77.9	85.3	89.3	78.3	84.2	89.6	84.2	89.9	92.8	81.5	87.0	71.7	87.0
Blood	1,236	87.9	93.0	95.9	87.8	91.7	95.7	91.5	95.5	97.4	90.7	93.6	81.5	93.6
Onset														
Hospital	6,445	76.2	84.7	89.7	76.1	82.9	89.2	82.2	89.2	92.8	82.6	87.3	71.2	87.3
Admission	5,256	82.4	87.8	90.4	83.3	87.6	91.5	88.4	92.1	93.9	82.2	88.2	74.6	88.2
Urban/rural														
Urban	11,176	78.6	85.8	89.8	79.2	84.9	90.1	84.6	90.2	93.1	82.3	87.5	72.5	87.5
Rural	525	86.7	93.1	94.5	82.1	88.0	93.9	91.6	95.8	97.1	86.1	91.4	77.0	91.4
Teaching status														
Nonteaching	6,904	77.5	84.8	89.1	77.4	83.7	89.0	83.6	89.6	92.5	81.2	86.9	71.2	86.9
Teaching	4,797	81.1	88.0	91.4	82.1	86.8	92.0	86.9	91.8	94.5	84.3	88.9	74.9	88.9
Bed size														
>300	8,216	76.9	84.8	88.9	77.4	83.8	89.1	83.3	89.4	92.4	81.5	86.9	72.1	86.9
100–300	3,092	83.9	88.9	92.8	83.8	87.7	92.9	88.9	92.8	95.3	84.7	89.3	73.8	89.3
<100	393	83.0	90.3	92.4	85.0	89.6	93.9	89.1	94.4	95.7	85.8	92.1	78.6	92.1
CDC region														
South	5,785	80.0	87.2	90.6	80.3	86.0	90.5	86.2	91.6	93.9	82.8	88.3	74.6	88.3
Midwest	3,293	77.7	85.3	88.9	77.8	83.8	89.3	83.8	89.7	92.3	80.4	86.4	71.1	86.4
West	1,428	77.7	83.4	90.5	78.9	82.9	90.7	80.8	86.5	92.7	84.9	88.4	70.6	88.4
Northeast	1,195	78.9	86.2	89.7	79.2	86.0	90.8	87.1	92.2	93.6	83.0	87.7	71.0	87.7

a AG, aminoglycoside (gentamicin, tobramycin, amikacin); Carb, carbapenem (imipenem, meropenem); CDC, Centers for Disease Control and Prevention; combo, combination antibiogram; ESC, extended-spectrum cephalosporins (ceftazidime, cefepime); FQ, fluoroquinolone (ciprofloxacin, levofloxacin); ICU, intensive care unit; TZP, piperacillin-tazobactam.

### P. aeruginosa susceptibility to antibiotic combinations.

Combinations of antibiotics with a backbone antibiotic and either an FQ or an aminoglycoside (AG) resulted in improved *in vitro* antibiotic coverage compared with that achieved with single agents (Table 2). The addition of an AG resulted in greater increases in antibiotic coverage (8.3% to 15%) than the addition of an FQ (5.2% to 7.1%). The antibiotic combination with the lowest susceptibility was a carbapenem (Carb) and an FQ (85.0%), and the combination with the highest susceptibility was piperacillin-tazobactam plus AG (93.3%). In approximately one-third of the 304 facilities in this analysis, adding a second agent (either an FQ or an AG) did not result in a notable improvement in susceptibility (a ≤1% increase with the combination versus that with single-agent therapy in 33.6%, 27.3%, and 38.5% of facilities for a Carb, an extended-spectrum cephalosporin [ESC], and piperacillin-tazobactam, respectively). Minimal increases in susceptibility (≤1%) with combination therapy compared with that with single-agent therapy were more common in facilities reporting 30 isolates or less.

Improvements in antibiotic coverage with combination therapy were similar for respiratory and blood isolates. For blood isolates, greater than 95% susceptibility was achieved for the ESC plus AG (95.9%), Carb plus AG (95.7%), piperacillin-tazobactam plus AG (97.4%), and piperacillin-tazobactam plus FQ (95.5%) combinations. None of the combinations resulted in >95% susceptibility for respiratory isolates or for combined blood plus respiratory P. aeruginosa isolates (Table 2).

In secondary analyses, we compared susceptibility rates in the primary data analysis set (presumed unsuppressed; *n* = 11,701 isolates), which included isolates for which susceptibility was not reported (inferred to be susceptible), with susceptibility rates for isolates with full antimicrobial susceptibility reporting (the complete antimicrobial susceptibility testing [AST] subset; *n* = 9,492 isolates) (Table S2). The difference between these two data sets was 2,209 isolates; accordingly, results based on the complete AST reporting subset were suppressed for approximately 20% of the isolates in the primary analysis data set (2,209/11,701 = 18.9%). Susceptibility rates for total and respiratory P. aeruginosa isolates were approximately 2% to 6% higher when isolates with suppressed antimicrobial susceptibility data were included (primary data set; presumed unsuppressed) than when the complete AST reporting subset was included, while susceptibility rates in blood isolates were similar for these two data sets.

In order to further explore the impact of selective antimicrobial susceptibility reporting, we examined data from which hospitals with more aggressive guidelines for suppression of antimicrobial susceptibility reporting were excluded. Our analyses focused on data from the subset of 237 hospitals reporting antimicrobial susceptibility to all 5 antimicrobial classes in >70% of P. aeruginosa isolates. Susceptibility rates based on presumed unsuppressed data from hospitals reporting antimicrobial susceptibility to all 5 antimicrobial classes in >70% of P. aeruginosa isolates (Table S3) were generally comparable to those based on primary data for presumed unsuppressed results from all hospitals (Table S2). Susceptibility rates from presumed unsuppressed results from all hospitals were slightly higher (2% to 3%) than the susceptibility rates from the complete AST reporting subset in hospitals reporting antimicrobial susceptibility to all 5 antimicrobial classes in >70% of P. aeruginosa isolates (Table S3). However, the difference between susceptibility rates from presumed unsuppressed results versus the complete AST reporting data was not as large in hospitals reporting antimicrobial susceptibility to all 5 antimicrobial classes in >70% of P. aeruginosa isolates (Table S3) as in the full data set with data from all hospitals (Table S2).

### Effects of ICU status on susceptibility.

**FIG 1:**
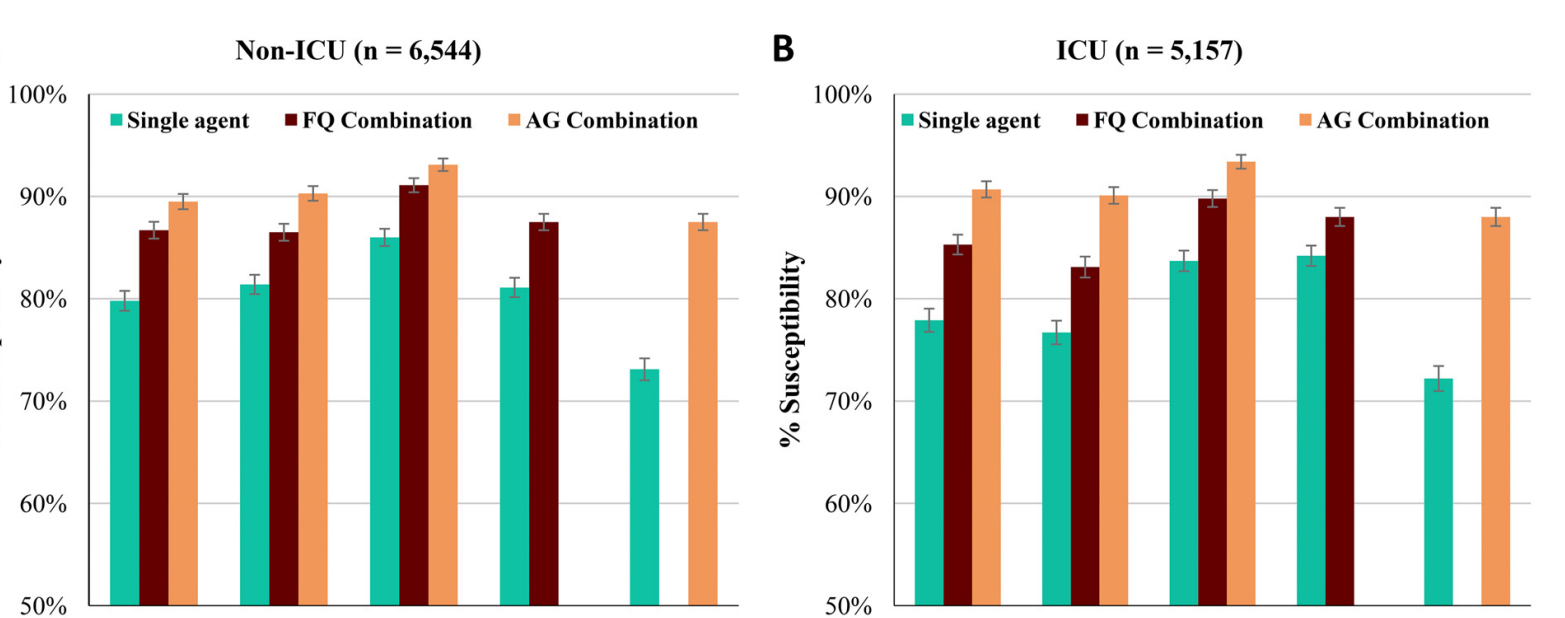
Antibiotic therapy coverage (mean percent susceptibility). (A) Non-ICU isolates; (B) ICU isolates. Capped error bars indicate 95% confidence intervals. ESC, extended-spectrum cephalosporins (ceftazidime, cefepime); Carb, carbapenem (imipenem, meropenem); TZP, piperacillin-tazobactam; AG, aminoglycoside (gentamicin, tobramycin, amikacin); FQ, fluoroquinolone (ciprofloxacin, levofloxacin).

We observed modest differences in mean susceptibility by ICU admission status (Fig. 1). Univariate (unadjusted) analysis found that non-ICU admission was associated with significantly higher susceptibility rates than ICU admission for the three beta-lactam agents (an ESC, a Carb, piperacillin-tazobactam) as either single agents or in combination with an FQ (Table 3). In contrast, non-ICU admission was associated with significantly lower susceptibility to AG as a single agent than ICU admission. ESC plus AG also showed significantly lower susceptibilities for non-ICU admission than for ICU admission, but none of the other AG combinations showed a significant difference. There were no significant differences in susceptibility between ICU and non-ICU admission for FQ alone or for an FQ in combination with an AG.

**TABLE 3:** Unadjusted and adjusted effects of ICU status on susceptibility^*a*^

Effect and antibiotic	Single agent			FQ combination			AG combination		
	OR	95% CI	*P*	OR	95% CI	*P*	OR	95% CI	*P*
Unadjusted effects for non-ICU vs ICU status									
ESC	1.12	1.02–1.22	0.0132	1.13	1.02–1.25	0.0241	0.88	0.78–0.99	0.0410
Carb	1.33	1.21–1.45	<0.0001	1.30	1.18–1.44	<0.0001	1.02	0.90–1.16	0.7211
TZP	1.20	1.08–1.33	0.0005	1.16	1.03–1.32	0.0162	0.95	0.82–1.10	0.5103
AG	0.81	0.73–0.89	<0.0001	0.95	0.85–1.06	0.3415	NA	NA	NA
FQ	1.05	0.96–1.14	0.2752	NA	NA	NA	0.95	0.85–1.06	0.3415
GLMM-adjusted effects^*b*^ for non-ICU vs ICU status									
ESC	1.06	0.96–1.17	0.2320	1.05	0.94–1.18	0.3987	0.83	0.72–0.94	0.0048
Carb	1.27	1.15–1.40	<0.0001	1.21	1.08–1.35	0.0009	0.96	0.84–1.09	0.5211
TZP	1.14	1.02–1.27	0.0217	1.07	0.94–1.23	0.3023	0.91	0.78–1.06	0.2324
AG	0.76	0.69–0.85	<0.0001	0.89	0.79–1.01	0.0683	NA	NA	NA
FQ	0.96	0.88–1.05	0.3954	NA	NA	NA	0.89	0.79–1.01	0.0683

a Numbers indicate the effect size of non-ICU status; i.e., an odds ratio of 1.12 for single-agent ESC indicates that the odds of a P. aeruginosa isolate being susceptible to ESC is 12% higher for non-ICU isolates than for ICU isolates. Abbreviations: AG, aminoglycoside (gentamicin, tobramycin, amikacin); Carb, carbapenem (imipenem, meropenem); CI, confidence interval; ESC, extended-spectrum cephalosporins (ceftazidime, cefepime); FQ, fluoroquinolone (ciprofloxacin, levofloxacin); GLMM, generalized linear mixed models; ICU, intensive care unit; NA, not applicable; OR, odds ratio; TZP, piperacillin-tazobactam.

b Effects were adjusted using GLMM. Adjusting variables include source (blood/respiratory), onset (admission/hospital), and hospital characteristics (teaching status, bed size, urban/rural, geographic region).

Some, but not all, of the findings in the univariate analysis were confirmed by the multivariable generalized linear mixed model (GLMM) analysis. As in the univariate analysis, susceptibilities to single-agent Carb and piperacillin-tazobactam were significantly higher in non-ICU patients than in ICU patients, and susceptibilities to single-agent AG and ESC plus AG were significantly lower (Table 3). The susceptibility to single-agent ESC was also higher in non-ICU patients than in ICU patients, but the effect was no longer statistically significant in the adjusted analysis. The only FQ therapy that maintained a significant effect for non-ICU versus ICU patients was Carb plus FQ.

## DISCUSSION

Although there are currently no CLSI guidelines for the use of combination antibiograms, our study suggests that this laboratory tool can provide important information concerning the empirical combination regimens most likely to be successful against P. aeruginosa at a given institution. It has been recognized for decades that combination antibiotic therapy has the potential to improve antimicrobial coverage through multiple mechanisms, including alternate mechanisms of action, synergistic effects, and reduced development of resistance (5, 13). Empirical combination therapy is particularly important for pathogens such as P. aeruginosa that exhibit high rates of resistance, often to multiple drugs (14). A recent U.S. study from the Program To Assess Ceftolozane-Tazobactam Susceptibility (PACTS) found that 23.6% of 1,576 P. aeruginosa respiratory isolates were multidrug resistant (nonsusceptible to at least 3 antimicrobial classes) and 9.8% were extensively drug resistant (nonsusceptible to at least one agent in all but two antimicrobial classes or fewer) (15). Similar results were observed in a PACTS study of P. aeruginosa isolates from various infection sites (bloodstream, lower respiratory tract [pneumonia], skin, urinary tract, and intra-abdominal region) in U.S. medical centers (783 of 3,737 [21.0%] isolates were multidrug resistant, and 9.3% were extensively drug resistant) (16) and an international multicenter study of P. aeruginosa nosocomial pneumonia (226 of 740 [30.5%] patients were infected with MDR strains) (17). Empirical combination therapy increases the likelihood of achieving appropriate therapy against P. aeruginosa (18), which may be particularly critical for ICU patients. A single-center study found that 75% of adult ICU patients with P. aeruginosa BSI received inappropriate initial therapy (19). Inappropriate (18, 20) or delayed appropriate (21) empirical antibiotic therapy is consistently associated with increased mortality in patients with P. aeruginosa BSI. In recognition of the high morbidity and mortality associated with P. aeruginosa infections, U.S. and international guidelines recommend empirical combination therapy with two antipseudomonal drugs for the treatment of HAP/VAP in patients with risk factors for multidrug resistance, with a goal of achieving >95% coverage of likely pathogens (10, 11).

Frequently, however, empirical combination therapy is based on knowledge of single-agent antibiograms; additional information to help guide selection of the optimal combination regimen is often lacking. Combination antibiograms provide a useful clinical tool for evaluating the breadth of antimicrobial coverage of multiple agents (22). For instance, a single-center study of combination antibiograms found that addition of any second agent (an AG or FQ) to a backbone beta-lactam (an ESC, a Carb, or piperacillin-tazobactam) significantly increased the proportion of P. aeruginosa isolates with adequate antimicrobial coverage (23), thus providing clinicians with information of potential relevance when choosing empirical therapy for severely ill patients.

In our study, neither single-agent antibiotics nor combinations of antibiotics, as assessed by combination antibiograms, achieved 95% *in vitro* coverage for P. aeruginosa respiratory and blood isolates. This observation suggests a need for the expanded use of newer approved systemic antimicrobials, the development of new antipseudomonal drugs, and the utilization of strategies to improve pharmacodynamics for serious P. aeruginosa infections (24).

The finding that susceptibility rates for piperacillin-tazobactam were higher than those for carbapenems is in contradiction to the findings of some other large surveillance studies, which have reported higher rates of susceptibility to carbapenems (15) or similar rates of susceptibility to piperacillin-tazobactam and carbapenems (16), based on CLSI breakpoints. For comparison, the 85.0% piperacillin-tazobactam susceptibility rate for P. aeruginosa reported here is higher than the 79.1% CLSI piperacillin-tazobactam susceptibility rate reported for U.S. P. aeruginosa isolates (*n* = 4,487 isolates from all infection sites) in 2016 and 2017 in the online SENTRY database (https://sentry-mvp.jmilabs.com/app/sentry-public) and the 71.2% CLSI piperacillin-tazobactam susceptibility rate reported for U.S. P. aeruginosa isolates (*n* = 896 isolates from intra-abdominal, urinary tract, and lower respiratory tract specimens) in the Study for Monitoring Antimicrobial Resistance Trends (SMART) database (25). It is possible that our findings reflect the widespread use of FDA susceptibility breakpoints for piperacillin-tazobactam in hospital laboratories (≤64 mg/liter, compared with ≤16 mg/liter for CLSI breakpoints), resulting in increased piperacillin-tazobactam susceptibility rates. In addition, carbapenem susceptibility rates may have been reduced by the inclusion of both imipenem and meropenem, as imipenem susceptibility rates for P. aeruginosa in patients with VAP tend to be lower than meropenem susceptibility rates (26). In our study, imipenem-nonsusceptible isolates would have been excluded from the carbapenem-susceptible category, even if the isolates were susceptible to meropenem, thus potentially reducing the rates of carbapenem susceptibility. It is also possible that the geographic distribution of hospitals in our study influenced our findings, as regional variations in P. aeruginosa susceptibility to different agents have been noted (15, 27).

We found that adding an AG to a backbone antibiotic resulted in higher susceptibility rates than the addition of an FQ, which is consistent with the results from single-center and regional studies of combination antibiograms (23, 28–30). Together, these data seem to suggest that AGs should play a larger role as combination therapy in the treatment of serious infections. However, because of the low level of lung penetration by AGs, it is possible that *in vitro* antibiograms do not reflect clinical outcomes in HAP/VAP. Both AGs and FQs have significant toxicities and the potential for adverse drug events, most notably, nephrotoxicity with AGs and Clostridium difficile infection with FQs (31). A combination antibiogram might help inform risk-benefit decisions for individual patients.

Selective reporting of antimicrobial susceptibility influences prescribing practices and has shown some success as an antibiotic stewardship measure (32, 33) but also limits the amount of information available to clinicians and potentially skews susceptibility rates. The CDC recommends that resistance rates be interpreted with caution if the percentage of isolates with susceptibility tests or reports is less than 70% (34). A CDC analysis of the most recent National Healthcare Safety Network data (2012 to 2014) noted declining percentages of reported susceptibility test results for some pathogen-antibiotic combinations; carbapenem susceptibility was reported for 76.5% to 81.7% of P. aeruginosa isolates, depending on the nosocomial infection (34). Similarly, in our study, P. aeruginosa susceptibility results were suppressed for approximately 20% of isolates. We found that presumed unsuppressed reports resulted in antimicrobial susceptibility rates approximately 2% to 6% higher than those in the complete AST reporting subset, suggesting that inferring susceptibility may slightly inflate susceptibility rates. More comparable rates were observed when the analyses were limited to hospitals reporting antimicrobial susceptibility in >70% of P. aeruginosa isolates. We consider it unlikely that these modest differences have a substantial effect on empirical antibiotic selection. However, on the basis of these data, we recommend that combination antibiogram analyses utilize unsuppressed data when possible, particularly in hospitals reporting susceptibility for fewer than 70% of isolates, in keeping with the aforementioned CDC recommendation (34).

In our study, blood isolates showed greater susceptibility than respiratory isolates. These findings are consistent with recent reports from U.S. medical centers, in which P. aeruginosa BSI isolates (*n* = 355) had higher susceptibility rates than pneumonia isolates (*n* = 1,576) to all antibiotics tested (15, 35).

We identified modest differences in susceptibility based on ICU status. In multivariable analyses, single-agent beta-lactam and FQ combinations were associated with significantly lower susceptibility rates in ICU than in non-ICU settings. ICU isolates are likely more resistant due to patient risk factors, including comorbidities, immunosuppression, foreign invasive devices, and prior antibiotic treatment (28). Somewhat surprisingly, however, susceptibility rates for a single-agent AG were higher in the ICU.

A limitation of this study is that combination therapy data are based on additive *in vitro* data for the specified drugs. Isolates were not tested against the actual combination *in vitro*, so synergy and interference were not evaluated. *In vitro* antimicrobial synergy against some isolates of *Pseudomonas* has been reported for combinations of an ESC with an AG or FQ (36). In addition, data were collected and analyzed from the perspective of unique nonduplicate collected cultures and not from the perspective of unique patients. We therefore do not have data on the clinical outcomes of patients treated with monotherapy versus combination therapy. Susceptibility data were provided by the participating hospitals and relied on the interpretive results reported at each facility. Not all hospitals in the full database submitted susceptibility data, and there were no uniform systems of testing or predefined breakpoints. For the primary analyses, we assumed that isolates were susceptible at an antibiotic class level if not reported as intermediate or resistant, which may have resulted in inflated susceptibility rates.

### Conclusions.

We draw two important conclusions from the data presented here: (i) combination antibiograms may be useful in evaluating local *in vitro* susceptibility, and (ii) from an *in vitro* susceptibility perspective, currently available drugs do not achieve the recommended coverage level against P. aeruginosa, even in combination, suggesting the need for new drugs to combat this pathogen.

Combination antibiograms have the potential to optimize empirical therapy of P. aeruginosa and may help inform institutional guidelines and pathways. Local institutions should consider utilizing combination antibiograms to determine local susceptibility data and assist in guiding empirical therapy in patients with serious infections, including suspected P. aeruginosa HAP/VAP or BSI. The need for local data is supported by our observation that there was no benefit in susceptibility rates (≤1% improvement) with combination therapy versus single-agent therapy at approximately one-third of the facilities in this study. For larger hospitals, combination antibiograms specific for certain hospital units (ICU, hematology/oncology, and others) may be required to tailor therapy to specific patient populations and pathogens. We encourage the development of CLSI guidelines for combination antibiograms to help promote the effective use of this valuable tool at the institutional level. We envision these future CLSI guidelines as providing guidance on methodologies for combination antibiograms and recommendations for key antibiotic combinations to be tested. A growing number of hospitals are starting to report antibiotic resistance data electronically to CDC’s Antimicrobial Resistance Option. If guidance for preparing combination antibiograms is developed, future iterations of the Antimicrobial Resistance Option could incorporate that guidance.

Although HAP/VAP guidelines recommend >95% coverage for likely pathogens, commonly used antibiotics, either alone or in combination, do not achieve this level of antimicrobial coverage for P. aeruginosa. The data from our study thus strongly support the need for new drugs or combinations of drugs to treat infections caused by P. aeruginosa. Possible candidates for effective antipseudomonal antibiotics include two recently approved beta-lactam–beta-lactamase inhibitor combinations, ceftolozane-tazobactam and ceftazidime-avibactam, and drugs in late-stage clinical development, such as cefiderocol and imipenem-relebactam (37, 38). Combination antibiogram analyses with these antibiotics may provide insights into their potential contribution to antipseudomonal therapy.

## MATERIALS AND METHODS

### Study design.

This was a retrospective cross-sectional study of the antimicrobial susceptibility of all nonduplicate (the first isolate in 30 days) P. aeruginosa blood and respiratory clinical isolates from ICU and non-ICU patients collected from 1 October 2016 to 30 September 2017. Isolates from blood and respiratory sources were considered separately; if the patient had a blood and respiratory isolate for P. aeruginosa within 30 days, then one isolate was counted for each source. Isolates collected within 30 days were included if they had different susceptibilities (>1 susceptibility difference). The objective of this study was to evaluate *in vitro* P. aeruginosa susceptibility rates to single-agent and combination antibiotic regimens through the use of a combination antibiogram in isolates obtained from respiratory and blood samples in ICU and non-ICU settings.

Reporting institutions for this analysis consisted of U.S. hospitals included in the BD Insights Research Database (Becton, Dickinson and Company, Franklin Lakes, NJ). The electronic surveillance system and clinical research database (formerly the CareFusion Clinical Research Database) have been previously described (39–41). This database provides good geographical representation across the United States and includes both small and large hospitals in urban and rural areas (see Table S1 in the supplemental material). Included hospitals were those reporting susceptibility results for all five of the specified antibiotic classes (see below). The study was approved by the New England Institutional Review Board (Wellesley, MA).

The primary analyses reported here included all nonduplicate P. aeruginosa isolates with facility-reported susceptibility results for at least one antibiotic in each of the five following antibiotic classes: (i) extended-spectrum cephalosporins (ESC; ceftazidime, cefepime), (ii) carbapenems (Carb; imipenem, meropenem), (iii) piperacillin-tazobactam, (iv) aminoglycosides (AGs; gentamicin, tobramycin, amikacin), and (v) fluoroquinolones (FQs; ciprofloxacin, levofloxacin). Isolates were considered susceptible to an antibiotic class level if they were reported to be susceptible or if they were not reported to be intermediate or resistant (i.e., isolates not reported to be intermediate or resistant were inferred to be susceptible to the antimicrobial class). By including isolates not specifically reported to be susceptible, the primary analyses thus provide presumed unsuppressed data; the numerator represents isolates with reported susceptibility plus isolates whose susceptibilities were not reported and that were presumed to be susceptible, and the denominator represents all isolates tested for susceptibility to any of the 5 antibiotic classes. Secondary analyses were performed to compare the primary data analysis set with isolates in which susceptibility to all antibiotics was reported without inference (complete antimicrobial susceptibility testing [AST] subset) from all hospitals reporting susceptibility to all 5 antimicrobials, as well as to compare the full data set (presumed unsuppressed) with the complete AST subset for isolates from hospitals reporting susceptibility to all 5 antimicrobials for >70% of P. aeruginosa isolates.

Nursing units were classified using the CDC National Healthcare Safety Network classification and further classified as ICU (critical care) and non-ICU (inpatient adult wards, specialty care areas, and step-down wards) (42). ICU categorization of isolates was based on the following criteria: (i) for patients admitted to the ICU within 3 days of inpatient admission and with no previous admission within 14 days, the isolate had to be collected in the ICU, and (ii) for patients admitted to the ICU >3 days after hospital admission, the isolate had to be collected in the ICU >3 days after the time of ICU admission (hospital onset, ICU acquired). All other hospital isolates were categorized as non-ICU. Hospital-onset infections were defined as those occurring >3 days after inpatient admission or within 14 days of previous discharge, while admission infections were defined as those occurring ≤3 days after inpatient admission with no previous admission within 14 days.

### Outcomes.

The primary outcomes were the rates of P. aeruginosa susceptibility to antimicrobial drug classes (an ESC, a Carb, piperacillin-tazobactam, FQs, and AGs) as single agents or in combinations of backbone antibiotics (an ESC, a Carb, and piperacillin-tazobactam) with an AG or an FQ. Antimicrobial susceptibility determinations were based on local laboratory breakpoints and practices. For antibiotic combinations, susceptibility was defined as susceptibility to at least one agent in the combination.

### Statistical analysis.

Statistical analysis, including descriptive analysis and statistical modeling, was conducted for each combination of the backbone antibiotics and antibiotic regimen (for example, an ESC as a single agent, an ESC and an AG in combination, an ESC and an FQ in combination, a Carb as a single agent, and a Carb and an AG in combination, etc.). In the univariate (unadjusted) analysis phase, we used chi-square tests (or Fisher’s exact tests for events with an expected frequency of <5) to examine the associations between outcome variables and potential predictors. The potential predictors or confounding variables included ICU admission status, source of isolate, isolate onset (admission or hospital onset), and hospital characteristics (teaching status, bed size, urban/rural, and geographic location). In the multivariable (adjusted) analysis phase, we used the generalized linear mixed model (GLMM) method with hospital as the random effect to assess the effect of ICU status on the susceptibility rate. Specifically, the outcomes were modeled using random-intercept logistic regression models with hospital as the random effect. All analyses were conducted using SAS (version 9.4) software (SAS Institute, Cary, NC).

## Supplementary Material

Supplemental file 1
