# Supplementary material for: A Combination Antibiogram Evaluation for Pseudomonas aeruginosa in Respiratory and Blood Sources from Intensive Care Unit (ICU) and Non-ICU Settings in U.S. Hospitals
Source: Antimicrob Agents Chemother. 2019 Mar 27;63(4):e02564-18. doi: 10.1128/AAC.02564-18 (PMC6496158; doi:10.1128/AAC.02564-18)
Supplement: Supplemental file 1 [file AAC.02564-18-s0001.pdf]

Supplemental Table S1. Hospital characteristics of BD Insights Research Database hospitals compared with Centers for Medicare & Medicaid Services (CMS) hospitals

	**BD Hospitals (n=304)**	**CMS (n=4,655)**
**Urban/Rural**		
Urban	82.9%	59.3%
Rural	17.1%	40.7%
**Teaching status**		
Non-teaching	62.8%	N/A
Teaching	37.2%	N/A
**Bed size**		
**<100**	18.1%	50.9%
**100-300**	45.7%	29.4%
**>300**	36.2%	19.7%
**US census region**		
Northeast	11.2%	12.3%
South	46.3%	37.9%
Midwest	29.3%	29.9%
West	13.2%	19.9%

Abbrevations: N/A not applicable.

Supplemental Table S2. Antimicrobial susceptibility rates with suppressed antimicrobial susceptibility reporting compared with unsuppressed reporting for hospitals reporting susceptibility to all 5 antimicrobials (n=304). Isolates considered susceptible for the primary data set (presumed unsuppressed susceptibility reporting) were those with reported susceptibility plus those that were not reported as intermediate or resistant (susceptibility unreported). Isolates considered susceptible for the complete antimicrobial susceptibility testing (AST) subset were those reported as susceptible; no inference was made for unreported isolates. The presumed unsuppressed susceptibility rates are the same as the primary analysis data in Table 2 of the main manuscript and are provided here again for ease of comparison.

**Antibiotic**	**Primary data set (presumed unsuppressed susceptibility) (Total n= 11701; Respiratory n=10456; Blood n=1236)**			**Complete AST reporting subset (Total n=9492; Respiratory n=8386; Blood n=1106)**		
	**Single agent**	**AG combo**	**FQ combo**	**Single agent**	**AG combo**	**FQ combo**
ESC						
All	79.0%	90.0%	86.1%	74.1%	87.7%	82.9%
Respiratory	77.9%	89.3%	85.3%	72.5%	86.7%	81.6%
Blood	87.9%	95.9%	93.0%	86.5%	95.4%	92.1%
Carb						
All	79.3%	90.2%	85.0%	74.5%	88.0%	81.5%
Respiratory	78.3%	89.6%	84.2%	72.9%	87.0%	80.3%
Blood	87.8%	95.7%	91.7%	86.3%	95.2%	90.8%
TZP						
All	85.0%	93.3%	94.3%	81.5%	91.7%	88.3%
Respiratory	84.2%	92.8%	89.9%	80.3%	91.0%	87.4%
Blood	91.5%	97.4%	95.5%	90.5%	97.1%	94.9%
AG						
All	82.5%		87.7%	78.4%		84.9%
Respiratory	81.5%		87.0%	76.9%		83.8%
Blood	90.7%		93.6%	89.6%		92.9%

FQ						
All	72.7%	87.7%		66.4%	84.9%	
Respiratory	71.7%	87.0%		64.7%	83.8%	
Blood	81.5%	93.6%		79.3%	92.9%	

Abbreviations: AG aminoglycoside (gentamicin/tobramycin/amikacin), *Carb* carbapenem (imipenem/meropenem), *combo* combination antibiogram, *ESC* extended-spectrum cephalosporins (ceftazidime/cefepime), *FQ* fluoroquinolone (ciprofloxacin/levofloxacin), *TZP* piperacillin/tazobactam

Supplemental Table 3. Antimicrobial susceptibility rates with unsuppressed antimicrobial susceptibility reporting compared with selective reporting for hospitals for isolates reporting susceptibility to all 5 antimicrobials and reporting data for >70% of PsA isolates (n=237). Isolates considered susceptible for the primary data set (presumed unsuppressed susceptibility reporting) were those with reported susceptibility plus those that were not reported as intermediate or resistant (susceptibility unreported). Isolates considered susceptible for the complete antimicrobial susceptibility testing (AST) subset were those reported as susceptible; no inference was made for unreported isolates.

Antibiotic	Primary data set with >70% of PsA isolates reported (presumed unsuppressed susceptibility) (Total n=9169; Respiratory n=8113; Blood n=1056)			Complete AST reporting subset with >70% of PsA isolates reported (Total n= 8532; Respiratory n=7507; Blood n=1025)		
	**Single agent**	**AG combo**	**FQ combo**	**Single agent**	**AG combo**	**FQ combo**
ESC						
All	78.5%	90.4%	86.0%	76.9%	89.7%	85.0%
Respiratory	77.3%	89.7%	85.1%	75.5%	88.8%	83.9%
Blood	87.8%	96.0%	93.0%	87.4%	95.9%	92.8%
Carb						
All	79.4%	91.0%	85.1%	77.8%	90.4%	84.0%
Respiratory	78.2%	90.4%	84.2%	76.5%	89.6%	82.9%
Blood	88.1%	96.0%	92.0%	87.7%	95.9%	91.7%
TZP						
All	85.0%	93.8%	90.7%	83.9%	93.4%	90.0%
Respiratory	84.2%	93.4%	90.1%	82.9%	92.8%	89.3%
Blood	91.6%	97.5%	95.5%	91.3%	97.5%	95.4%
AGs						
All	82.3%		87.8%	80.9%		86.9%
Respiratory	81.1%		87.0%	79.6%		86.0%
Blood	90.8%		93.8%	90.5%		93.6%
FQ						
All	70.9%	87.8%		68.7%	86.9%	
Respiratory	69.5%	87.0%		67.1%	86.0%	
Blood	81.1%	93.8%		80.5%	93.6%	

Abbreviations: AG aminoglycoside (gentamicin/tobramycin/amikacin), *Carb* carbapenem (imipenem/meropenem), *combo* combination antibiogram, *ESC* extended-spectrum cephalosporins (ceftazidime/cefepime), *FQ* fluoroquinolone (ciprofloxacin/levofloxacin), *TZP* piperacillin/tazobactam
